# The Many Faces of Serous Neoplasms and Related Lesions of the Female Pelvis: A Review

**DOI:** 10.1097/PAP.0000000000000334

**Published:** 2022-02-18

**Authors:** Sameera Rashid, Maria A. Arafah, Mohammed Akhtar

**Affiliations:** * Department of Laboratory Medicine and Pathology, Hamad Medical Corporation, Doha, Qatar; † Department of Pathology, College of Medicine, King Saud University, Riyadh, Saudi Arabia

**Keywords:** fallopian tube carcinoma, ovarian carcinoma, pathogenesis of ovarian tumors, serous carcinoma, serous precursor lesions

## Abstract

Ovarian serous tumors and related lesions are one of the most common conditions of the female genital tract. While ovarian high-grade serous carcinoma carries high mortality and adverse prognosis, most other serous lesions have better clinical behavior. In recent years, significant progress has been made in understanding the nature and histogenesis of these lesions that has contributed to better and more precise clinical management. Most of the high-grade serous carcinomas involve the ovaries and/or peritoneum, although in most cases, their origin seems to be in the fallopian tube. This view is supported by the recognition of precursor lesions in the fallopian tube, such as p53 signature and serous tubular in situ carcinoma. This paper presents salient morphologic, immunohistochemical, and molecular data related to serous tumors and related lesions of the female pelvis and discusses the histogenetic interrelationship among these lesions in light of current knowledge.

Serous tumors and related lesions represent a group of common benign and malignant conditions involving the female pelvis (Table 1). These tumors are composed of epithelium with varying degrees of resemblance to the lining epithelium of the fallopian tube. Over the years, there has been much confusion and controversy regarding the origin and classification of these tumors. However, in recent years understanding of the origin and histologic characteristics has improved significantly as new concepts have emerged regarding their histogenesis. Furthermore, immunohistochemistry (IHC) and molecular testing have substantially improved the accuracy and reproducibility of these diagnoses. The recently published World Health Organization (WHO) classification has incorporated the current knowledge and understanding of these tumors.^1^

**TABLE 1:** Serous Neoplasms and Related Lesions of the Female Pelvis

Non-neoplastic lesions
Endosalpingiosis (fallopian tube–type epithelium)
Endometriosis (endometrial-type glands)
Endocervicosis (endocervical-type glands)
Benign neoplasms (p53 wild-type)
Surface papilloma
Serous cystadenoma
Serous cystadenoma with focal proliferation (proliferation <10%)
Serous adenofibroma
Serous cysdenofibroma
Borderline neoplasms (p53 wild-type)
Serous borderline tumor: epithelial proliferation >10% with no stromal invasion
Variants
Micropapillary/cribriform subtype: ≥5 mm of micropapillary/cribriform pattern
Microinvasion subtype: stromal invasive < 5 mm in single focus
Low-grade serous carcinoma (LGSC) (p53 wild-type)
Invasive serous carcinoma with low-grade nuclear morphology (<3-fold variation in nuclear size)
Variants
Microinvasive: serous borderline tumor with >5 mm invasive component
Invasive serous extraovarian implants
Microinvasive: LGSC morphology with stromal invasion <5 mm
High-grade serous carcinoma (HGSC) (p53-mutated)
Serous tumor with markedly atypical nuclei (>3-fold nuclear variability) and high mitotic rate
Extrauterine HGSC
Precursor lesion: serous tubal intraepithelial carcinoma
Homologous recombination-deficient tumors
SET pattern (solid, endometrial, transitional)
Uterine HGSC
Precursor lesion: serous endometrial intraepithelial carcinoma
Mixed carcinoma: HGSC with at least 1 additional pattern

## NON-NEOPLASTIC LESIONS: ENDOSALPINGIOSIS, ENDOMETRIOSIS, AND ENDOCERVICOSIS

**FIGURE 1:**
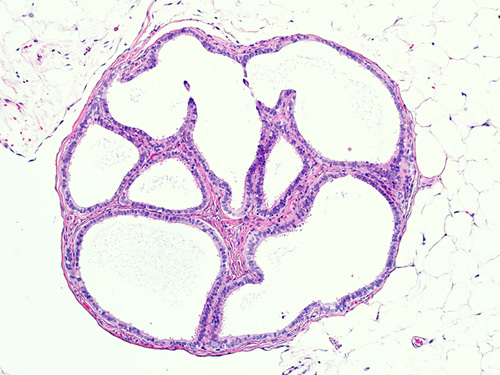
Endosalpingiosis in omental tissue characterized by multiloculated cystic structure lined by ciliated cuboidal epithelium.

**FIGURE 2:**
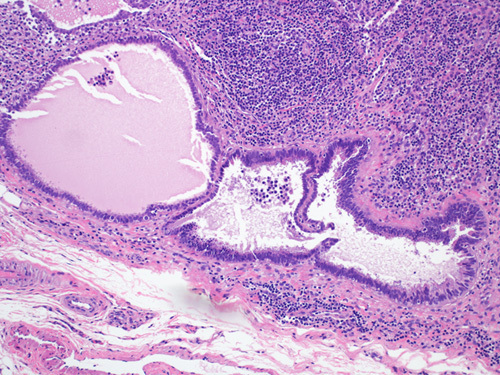
Endosalpingiosis in the lymph node can be challenging especially in cases where lymph nodes are removed because of a gynecologic malignancy. Notice the bland ciliated epithelium without complexity or atypia.

Endosalpingiosis is the presence of ectopic glands or cysts lined by fallopian tube–type ciliated epithelium (Fig. 1). The prevalence increases with age, especially after menopause. Endosalpingiosis may be found in pelvic organs, including ovaries, fallopian tube serosa, uterine serosa, myometrium, pelvic peritoneum, urinary bladder, or in retroperitoneal lymph nodes (Fig. 2). An unexplained feature of endosalpingiosis is its association with pelvic serous neoplasms, such as lesions of low malignant potential and low-grade pelvic serous carcinoma. However, the significance of endosalpingiosis as a risk factor or its role in the pathogenesis of these conditions is unknown.^2^–^5^ Endosalpingiosis can occur as an isolated pathologic finding, or it can be associated with foci of endometriosis or endocervicosis. Endometriosis is characterized by the presence of endometrial glands and stroma in an extrauterine location. It usually involves the pelvic organs but may also be present in a multitude of distant locations. In cases of endocervicosis, there is an endocervical type of glandular differentiation. It is primarily encountered in the urinary bladder, but it can rarely show other sites of involvement like axillary lymph nodes, rectum, vagina, cervix, and small intestine.^6^,^7^

## BENIGN SEROUS NEOPLASM: SEROUS CYSTADENOMA, ADENOFIBROMA, AND SURFACE PAPILLOMA

Benign ovarian serous tumors encompass several entities, including cystadenoma, adenofibroma (or cystadenofibroma), and surface papilloma. These tumors usually occur in adults with a wide age range varying from 40 to 60 years.^8^ They are bilateral in around 20% of cases.^8^,^9^

**FIGURE 3:**
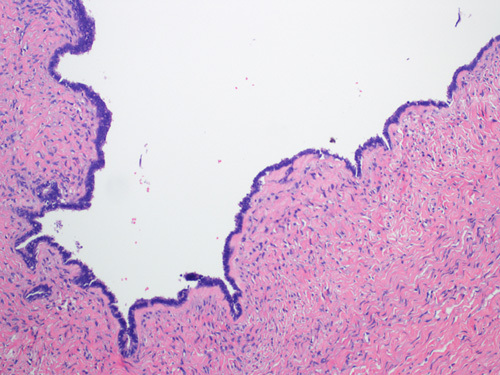
Microscopic picture of serous cystadenoma lined by a single layer of low cuboidal ciliated epithelium.

Serous cystadenomas are cystic lesions ≥1 cm in diameter, an arbitrary cutoff to distinguish them from the more common epithelial inclusion cysts. The lining epithelium consists of either a single layer of cuboidal cells (or flat attenuated cells in larger cysts) or more commonly pseudostratified cells resembling the lining of a fallopian tube and including a mixture of secretory cells with elongated nuclei and ciliated cells with rounded nuclei. The stroma surrounding the epithelium is usually fibrotic or edematous (Fig. 3). Psammoma bodies may be present, but nuclear atypia, mitoses, and cellular proliferation are absent.^1^,^8^ However, focal epithelial proliferation with epithelial stratification or detachment of cellular clusters may be seen. Cysts with proliferative components constituting <10% of the epithelium are called serous cystadenoma with focal proliferation.^9^

**FIGURE 4:**
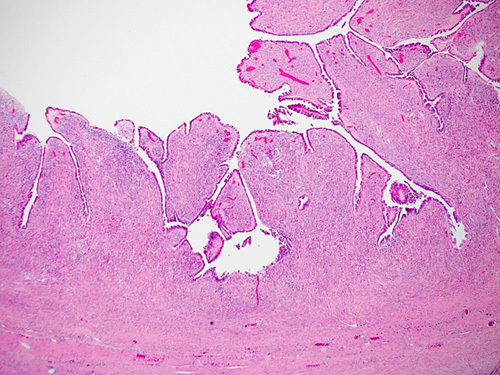
Histologic image of a serous cystadenofibroma with papillary luminal projections lined by pesudostratified ciliated cells and underlying fibrous stroma.

Serous adenofibroma shows papillary projections or scattered glands lined by serous epithelium and embedded in a more fibrotic and cellular stroma which commonly forms large solid areas (Fig. 4). When the lesion is also cystic, then the term “cystadenofibroma” is used.

Surface papillomas (or papillomatosis when multiple) consist of small papillary projections on the ovarian surface resembling adenofibromas or cystadenofibromas.

Most serous cystadenomas with limited epithelial proliferation may be non-neoplastic and best considered dilated or multilocular inclusion cysts.^1^ Similarly, most adenofibromas may be ovarian fibromas in which epithelial elements such as glandular inclusions or surface adhesions with a mesothelial lining are incorporated into the proliferating ovarian stroma.

The epithelium of benign serous ovarian tumors is positive for the common epithelial markers, including cytokeratin, epithelial membrane antigen, and MOC-31. They are also reactive for estrogen receptor (ER), PAX8, WT1, and p63.^9^,^10^ There is no aberrant p53 protein expression in benign serous ovarian tumors, and the Ki-67 proliferation index is usually low.^11^–^15^ Furthermore, p53 regulates the expression of p21, a cyclin-dependent kinase inhibitor, which is strongly expressed in benign ovarian serous tumors compared with borderline or malignant serous neoplasms.^16^ Nuclear overexpression for the c-Myc protein was found in 3 of 5 cases.^17^ Another study evaluated fascin protein expression and found that all serous cystadenomas were negative except for 4 of 20 cases that showed scattered epithelial cells staining (<10%).^18^

The epithelial lining in the benign serous neoplasm is usually polyclonal, although rare tumors may be monoclonal.^19^ A study by Cheng et al^15^ found that 25 of 29 (86%) serous cystadenomas were polyclonal, thus supporting the non-neoplastic nature of these lesions. Changes in DNA copy number have been reported in the epithelium of some benign serous cystadenomas (2.9%).^19^ Interestingly, clonal DNA aberrations were found more frequently in the fibromatous component of benign serous cystadenomas (33.3%), supporting the hypothesis of Seidman and Mehrotra that most of these tumors are probably primary fibromas.^19^ Benign ovarian serous tumors do not harbor the *KRAS* or *BRAF* mutations seen in borderline serous tumors or low-grade serous carcinomas (LGSCs).^15^,^19^

## BORDERLINE SEROUS TUMORS (ATYPICAL PROLIFERATIVE SEROUS TUMORS)

Serous borderline tumors occur in women with a mean age of 50 years, although a Chinese study of 225 patients found that the mean age is 32 years suggesting geographical variations. Around 37% to 55% of borderline serous tumors are bilateral.^9^,^20^,^21^

### Histopathologic Features

**FIGURE 5:**
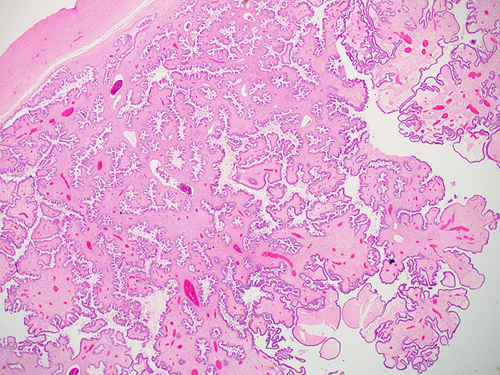
Photomicrograph of an ovarian tumor showing papillary projections with hierarchical branching and epithelial proliferation with stratification, lined by cells with low-grade atypia consistent with a serous borderline tumor.

**FIGURE 6:**
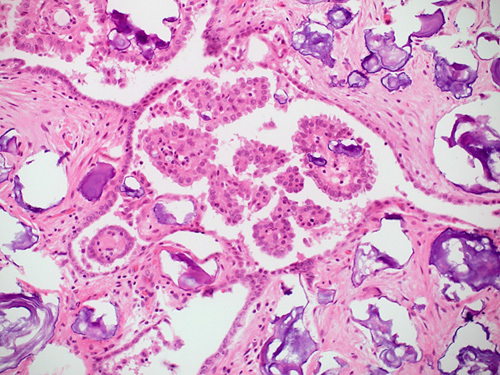
Serous borderline tumor with extensive Psammoma bodies. Notice the concentric lamellations in the Psammoma bodies.

Borderline serous tumors are composed of papillae with hierarchical branching and epithelial proliferation with stratification, budding, and detachment in at least 10% of the neoplasm (Fig. 5).^1^ The large central papillae contain variable amounts of stroma with fibrovascular cores and Psammoma bodies in almost half of the cases (Fig. 6).^9^ Several types of cells, including ciliated cells, hobnail-type cells, and eosinophilic cells with moderate cytoplasm and rounded nuclei, are usually present at the tips of the papillae or as free-floating clusters or as single cells.^20^ The nuclei show mild to moderate atypia and occasional mitoses (rarely exceeding 4 in 10 HPFs).^9^

**FIGURE 7:**
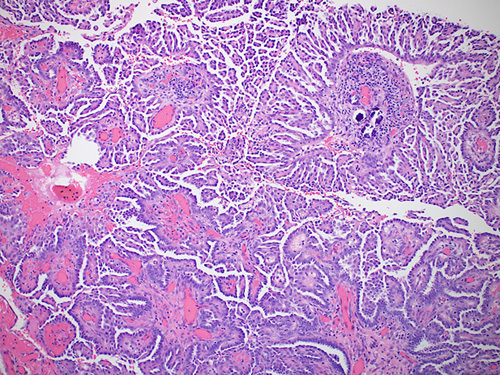
Photomicrograph showing nonhierarchical branching of filiform papillae arising from larger papillae (medusa-head appearance) in a serous borderline tumor with micropapillary pattern.

Some tumors manifest a micropapillary growth pattern characterized by nonhierarchical branching of elongated filiform papillae without fibrovascular cores (≥5 times longer than wide) emanating from larger fibrotic/edematous papillae (medusa-head appearance) (Fig. 7). Other tumors show a fusion of epithelial buds forming roman bridges or cribriform areas. Tumors in which micropapillary and cribriform patterns comprise ≥10% or measure ≥5 mm are classified as micropapillary type borderline serous tumors.^1^,^8^,^9^,^22^ The nuclear atypia in this variant may be slightly higher than the conventional type of borderline serous tumors. However, it still does not show high degree of atypia or pleomorphism characterizing high-grade serous carcinoma (HGSC). This variant accounts for 5% to 15% of ovarian serous borderline tumors and has a higher propensity for bilaterality, surface involvement, and implants.^23^,^24^ The term noninvasive LGSC was used for such lesions in the past. This, however, is now discouraged by the WHO.^1^

### Microinvasive Borderline Serous Tumors

**FIGURE 8:**
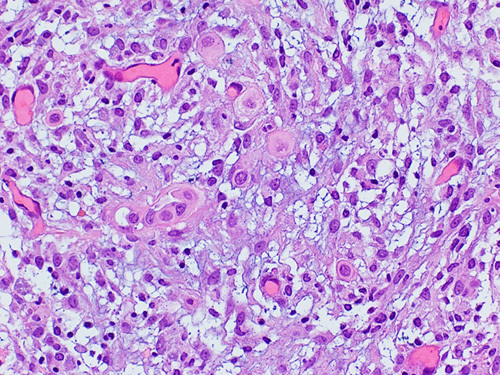
Serous borderline tumor with stromal microinvasion characterized by single eosinophilic cells.

**FIGURE 9:**
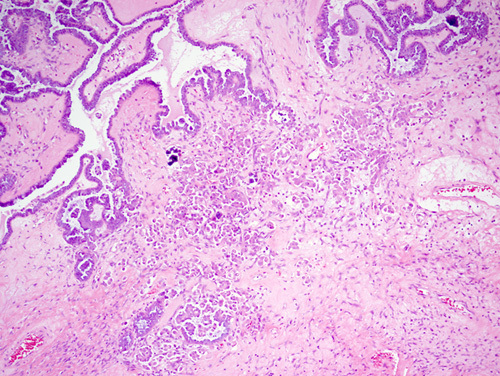
Microinvasive serous borderline tumor with glandular invasion of the underlying stroma.

Microinvasion in ovarian borderline serous tumors is defined as the presence of 1 or more foci of stromal invasion, each measuring <5 mm in the greatest dimension.^1^ It occurs in 10% to 15% of borderline serous tumors with a markedly increased incidence during pregnancy.^25^ In up to 60% of cases, the microinvasion may present as a lymphovascular invasion.^26^ Two types of microinvasion are recognized: the eosinophilic-cell type and the infiltrative type; the latter is more frequent in LGSCs (Figs. 8, 9).^27^

The eosinophilic-cell type of microinvasion comprises single cells or small clusters of eosinophilic cells budding into the stroma without invoking a desmoplastic reaction. The infiltrative type consists of nests of tumor cells or micropapillae resembling LGSC invading adjacent stroma. The invasive foci are surrounded by a clear space (a tissue retraction artifact) and a desmoplastic reaction.

### Implants

Implants are noninvasive extraovarian lesions associated with approximately one third of borderline serous tumors.^23^ They include 2 morphologic variants: epithelial and desmoplastic.

**FIGURE 10:**
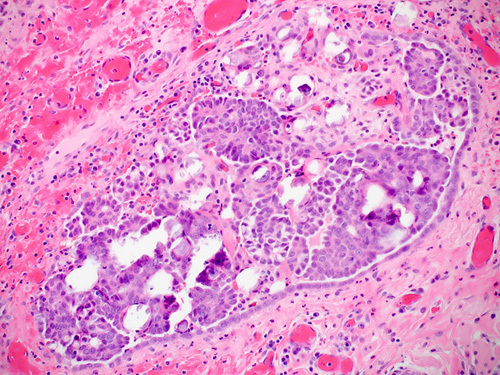
Photomicrograph of an epithelial implant showing a well-circumscribed lesion with papillae resembling serous tumor. Psammoma bodies can be identified.

Epithelial implants are relatively well-circumscribed and composed of papillae with hierarchical branching and fibrovascular cores resembling those seen in borderline serous tumors (Fig. 10). They are present on peritoneal surfaces and within the fibrous septa of the omentum without infiltrating the underlying tissue or fat lobules. The epithelium to stroma ratio is higher in epithelial implants than in desmoplastic implants.

**FIGURE 11:**
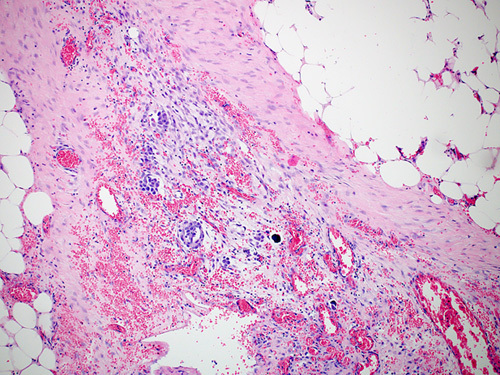
Histologic picture showing omental tissue with atypical cell clusters in a fibroblastic stroma consistent with a desmoplastic implant.

Desmoplastic implants are well-demarcated plaque-like thickenings overlying the peritoneal surface. These consist of exuberant fibroblastic proliferation and capillaries, surrounding papillae or glands, parallel or perpendicular to the peritoneal surface (Fig. 11). In addition, eosinophilic epithelial cells (similar to those seen in eosinophilic-cell type microinvasion) may be present in the stroma, scattered as single cells or arranged in small clusters. They are not considered invasive foci, provided they do not extend into the underlying fibrofatty tissue.^1^,^8^ Desmoplastic implants on the ovarian surface are also called “autoimplants.”^1^

The epithelial nuclear atypia in epithelial and desmoplastic implants is usually mild with rare mitoses. However, inflammation and Psammoma bodies are commonly present.

**FIGURE 12:**
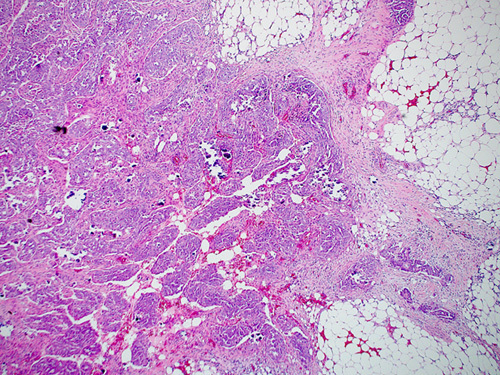
Microscopic image of an omental implant showing serous low-grade tumor invading the omental tissue, consistent with low-grade serous carcinoma. These were previously called invasive implants.

The previously known invasive implants are now considered invasive LGSC, and their recognition is the most important prognostic indicator of the disease. They are more frequent in micropapillary variants of borderline serous tumors. Among patients with peritoneal invasive LGSC “invasive implants,” 50% have micropapillary type serous borderline tumors, and 8% have conventional serous borderline tumors.^28^ Invasive LGSC show extensive epithelial proliferation with a haphazard infiltration to the underlying peritoneal fat (Fig. 12). The cells may show a confluent or a cribriform growth pattern or nests and micropapillae surrounded by a clear space or cleft (retraction artifact). The nuclei may show mild to moderate atypia with occasional mitoses. Noteworthy, in the presence of peritoneal invasive LGSC and an ovarian serous borderline tumor, the latter should not be classified as invasive LGSC.^10^

### Lymph Nodes Involvement

Forty-two percent of patients with LGSC have lymph node involvement beyond endosalpingiosis. The lymph node involvement may consist of eosinophilic cells present individually or in small clusters, mainly in the subcapsular sinuses. Another form consists of glands and papillae resembling LGSC within the node or just beneath the capsule. Although many of these lesions are not equivalent to metastatic carcinoma, they are staged as N1 disease.^1^,^29^ Lymph node involvement is usually associated with implants, but it does not have an independent prognostic significance. Lymph nodes with more pronounced epithelial proliferation packing the sinuses and haphazardly infiltrating the underlying lymph node parenchyma are classified as metastatic LGSC.

### Immunohistochemical Profile

Borderline serous tumors and LGSC express CK7, epithelial membrane antigen, BCL-2, ER, and progesterone receptor (PR). WT1 can be weak, and the Ki-67 index is usually low. Focal staining with CK20 may be seen in a few cases.^9^,^22^ Interestingly, microinvasive foci may show lower intensities of WT1, ER, PR, and Ki-67 staining, suggesting terminal differentiation or senescence in these cells.^30^ In one series, 46% of ovarian serous borderline tumors showed altered BRCA1 expression.^31^ Another study reported GLUT1 and p63 expression in 100% and 85% of borderline serous tumors, respectively.^32^ Fascin expression may be observed in 65% of borderline serous tumors.^18^

### Molecular Alterations

The ovarian serous borderline tumors are characterized by the mutually exclusive *KRAS* and *BRAF* mutations, each occurring in about 30% of borderline serous tumors.^23^ LGSC does not show microsatellite instability but may manifest DNA copy number changes.^33^

## LOW-GRADE SEROUS CARCINOMA

**FIGURE 13:**
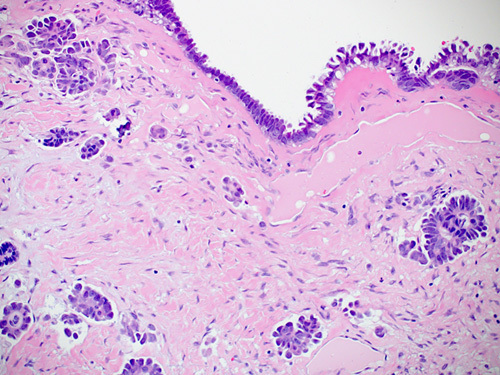
Section of an area of invasion in a serous borderline tumor amounting to >5 mm and consistent with a low-grade serous carcinoma.

**FIGURE 14:**
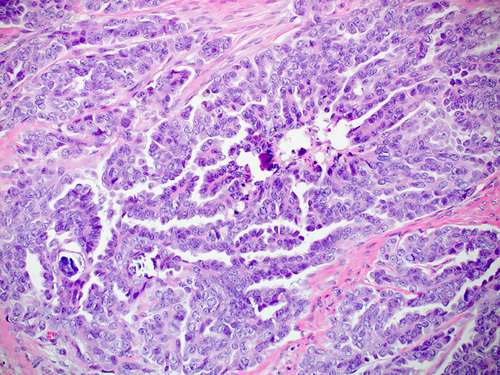
Low-grade serous carcinoma with papillary structures lined by ciliated cells with low-grade nuclear atypia. A Psammoma body can be identified in the lower left half of the picture.

LGSC is found in the ovary and is characterized by low-grade malignant serous cells in small nests, glands, papillae, micropapillae, or inverted micropapillae with cytologic atypia (<3-fold nuclear size variation) (Figs. 13, 14). Serous borderline tumors with stromal invasion >5 mm are also regarded as LGSC. Previously, serous borderline tumors with confluent micropapillary pattern >10% or 5 mm were regarded as noninvasive LGSC. This terminology is no longer recommended, and by definition, serous carcinoma represents invasive serous tumor. In rare instances, if the tumor has classic LGSC morphology but the area of invasion measures <5 mm, this is regarded as microinvasive LGSC with a recommendation for thorough sampling.

LGSC has no age predilection and is often bilateral. A high proportion of cases arise in a background of serous borderline tumor and carry *KRAS*, *NRAS*, and *BRAF* mutations similar to serous borderline tumors. In contrast to HGSC, LGSC has low mitosis, wild-type p53 expression, and patchy staining for p16. It is diffusely positive for ER, PR, CK7, and WT1.

LGSC is regarded as a distinct cancer type with different pathogenesis than HGSC. The clinical behavior of the 2 tumor types, as well as their pathogenesis, is discrete. LGSC has an indolent clinical course. Ovary-confined LGSC has an excellent prognosis while it is poor for patients with stages III-IV disease.^1^

## HIGH-GRADE SEROUS CARCINOMA

**FIGURE 15:**
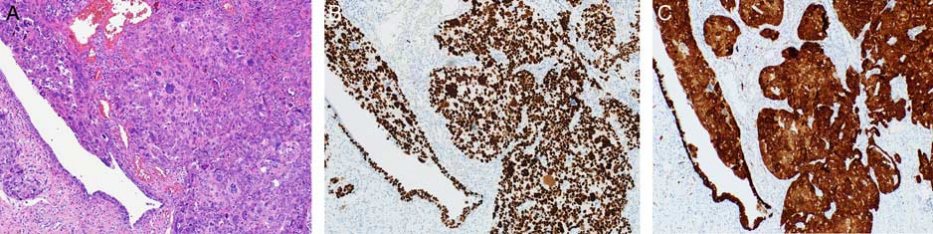
High-grade serous carcinoma. A, The tumor shows nuclear pleomorphism, increased atypical mitoses and hyperchromasia. Notice the bizarre nuclei that are a characteristic of a high-grade serous carcinoma. B, Immunohistochemical staining for p53 in a high-grade serous carcinoma showing strong positive staining in >90% of tumor cells. C, Immunohistochemical staining for p16 in a high-grade serous carcinoma showing block positivity.

HGSC manifests as complex papillae, sheets, or cords of cells with high-grade nuclei and marked pleomorphism (Fig. 15). In addition, there are abundant mitoses, slit-like spaces, and bizarre nuclei. Tufting, detached cell clusters, labyrinthine pattern, thick papillae, Psammoma bodies, and necrosis are also frequently identified. Areas with varying morphology from endometroid, transitional, mucinous, to even clear cell features can be observed. Unlike other ovarian epithelial neoplasms, HGSC does not require stromal invasion for diagnosis.^1^

Ovarian HGSC, primary peritoneal HGSC, and primary fallopian tube HGSC are collectively referred to as extrauterine pelvic serous carcinomas. In the uterus, it represents ∼10% of the endometrial cancers.

Among cancers of the female genital tract, ovarian HGSCs have the highest mortality.^34^ Two third of advanced-stage cases involve both ovaries, and nearly all involve the pelvic peritoneum (stage II) and serosa of bowel and/or other abdominal organs (stage III).

**FIGURE 16:**
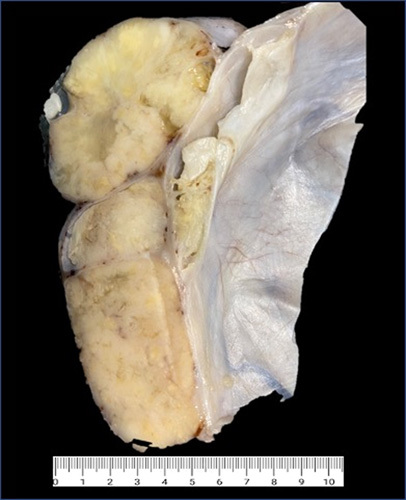
Gross photomicrograph of ovarian high-grade serous carcinoma. This is a multiloculated cyst with tan solid and cystic areas with focal necrosis. Sampling of the solid area is vital for diagnosis.

HGSC accounts for >75% of ovarian carcinomas. It is commonly seen in early postmenopausal women, and up to 20% are part of genetically inherited cancers. Ovarian HGSC commonly presents at an advanced stage with abdominal swelling and/or mass (Fig. 16). Hence, these are often treated with neoadjuvant chemotherapy.^35^,^36^

Invasion patterns have been studied extensively in many tumors, including cervical, endometrial, and ovarian mucinous carcinoma. However, this has only rarely been investigated in ovarian HGSC. Invasion patterns may be of 2 types: pushing or destructive. Others have recognized 3 types of invasion, namely, micropapillary, intracystic, and nonpapillary. Limited data show that the infiltrative (destructive) type of invasion is associated with aggressive tumor behavior and poor patient outcome.^37^

The Cancer Genome Atlas (TCGA) study showed that up to 96% of HGSC have *TP53* mutation, and *BRCA1/2* genes are involved in a significant number of HGSC, regardless of the germline status. It also found that almost half of the HGSC contained a mutation in a gene related to homologous recombination function.^15^,^38^

BRCA1 is a component of a complex molecule that surveys the genome for double-strand breaks. BRCA2, on the other hand, is involved in the repair by assisting the RAD51 complex. Hence, patients with germline mutations in *BRCA1/2* are at a higher risk for certain cancers than the general population. The risk of ovarian cancer in *BRCA1* and *BRCA2* mutation patients is 44% and 17%, respectively, whereas the lifetime risk of breast cancer is up to 72% in these patients.^39^

HGSC in patients with *BRCA1/2* mutations frequently show solid, endometrioid, and transitional cell-like areas termed SET features (solid, pseudoendometrioid, and transitional cell carcinoma-like).^40^ The SET variant, along with *BRCA* dysregulation and T-cell infiltration, has shown a favorable prognosis. In contrast, *CCNE1* amplification and reversion of *BRCA1/2* mutation may lead to treatment resistance in HGSC.^41^

p53 IHC is a reliable surrogate marker for *TP53* mutation.^42^ However, the interpretation of the p53 stain differs from other IHC stains. p53 IHC is interpreted as either mutated or wild-type instead of the traditional interpretation of positive and negative. Wild-type p53 expression in normal tissue manifests as focal weak heterogeneous nuclear staining where some cells show positive staining, some are negative, and the rest show different nuclear staining intensities. HGSC usually shows block positivity for p16 (Figs. 15A–C).

*TP53* mutation is associated with 4 p53 IHC staining patterns: overexpression, null pattern, cytoplasmic staining, and wild-type staining. Overexpression is the most common pattern observed in HGSC. It manifests as strong nuclear staining in 80% of the tumor cells. The null pattern of staining, seen in around 25% of HGSC, has no staining in tumor nuclei. The cytoplasmic staining pattern is seen in only 4% of the tumors and manifests as predominant cytoplasmic staining in the absence of strong nuclear staining in >80% of tumor cells. This pattern results from the disruption of the nuclear localization domain for *TP53* with an accumulation of p53 in the cytoplasm. The most challenging scenario is the wild-type pattern of staining in histologically HGSC, seen in around 5% of cases resulting from truncating *TP53* mutation manifested only after molecular testing.^42^,^43^ Highly proliferative nonserous carcinomas may show a high expression of p53 immunostain. This, however, should not be confused with overexpression of p53. A thorough examination in such cases shows <80% of the tumor cells with p53 staining. Heterogenous staining is another issue rarely encountered in large tumors and characterized by >1 staining pattern. This pattern is most likely due to autolysis and poor fixation.^43^

Around 40% of the p53 wild-type HGSCs have focal LGSC-like morphology and/or *RAS/RAF* mutation. These cases could represent a high-grade transformation in LGSCs. The prognostic significance of the p53 wild-type is unknown because of the limited data.^44^

Intermediate-grade serous carcinoma is a rarely used term in the literature for serous carcinoma with architectural features of LGSC but with focal high nuclear grade and intermediate mitotic index. Regardless of the *TP53* mutation status, the long-term survival in patients with these tumors may be similar to the classic HGSC group.^45^

Despite considerable research in tubo-ovarian HGSC and the use of different treatment strategies, the overall survival has not improved significantly. Optimum debulking and platinum sensitivity is associated with improved long-term survival.^46^ Patients with germline or sporadic *BRCA1/2* mutations not only have a good prognosis but are also targetable by poly (ADP-ribose) polymerase (PARP) inhibitors. Their use has increased disease-free survival in recurrent advanced-stage tumors.^47^

### Homologous Recombination Deficiency

BRCA1/2 genes are the most well-known mechanisms of homologous recombination deficiency. However, other mechanisms, such as germline and somatic mutations in other homologous recombination genes and epigenetic modifications, have also been implicated in homologous recombination deficiency. Ovarian cancer patients with homologous recombination deficiencies exhibit improved responses to treatments, such as platinum-based chemotherapy and poly (ADP-ribose) polymerase (PARP) inhibitors.^48^

### Therapy-related Effects

As most cases of ovarian HGSC present with extraovarian disease, interval debulking is frequently done after chemotherapy. These specimen usually show Psammoma bodies, hemorrhage, fibrosis, necrosis, and lymphocytic infiltrate. Bohm and colleagues proposed a 3-tier Chemotherapy Response Score (CRS) based on the quantity of residual cancer and associated response in the omentum. According to this system, CRS score 3 was defined as the lack of residual tumor or small foci of cancer up to 2 mm, and this was shown to be associated with better prognosis. CRS score 1 corresponds to no or minimal regression-associated fibroinflammatory response limited to a few foci. A score of CRS2 is assigned when there is an appreciable quantity of tumor with extensive fibroinflammatory change. These criteria are highly reproducible and associated with progression-free survival and overall survival.^47^

### Uterine Serous Carcinoma

**FIGURE 17:**
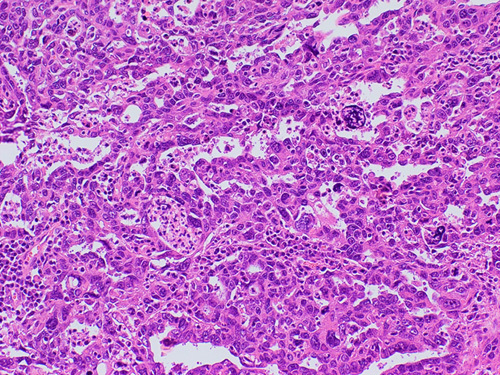
Histologic image of endometrial serous carcinoma with a basophilic look on histologic examination because of the high-grade atypia and bizarre nuclei.

**FIGURE 18:**
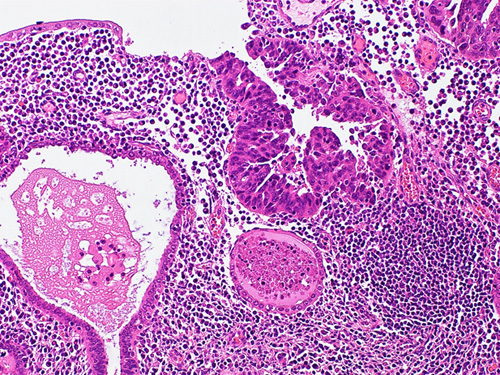
Histologic image of serous endometrial intraepithelial carcinoma. The endometrial glands show high-grade dysplasia in the background of atrophic glands with cystic change.

Uterine serous carcinoma (USC) accounts for ∼10% of all uterine carcinomas but represents a disproportionately higher number of uterine cancer-related deaths (80%).^49^ At presentation, around 37% to 70% of women have extrauterine spread.^50^,^51^ Microscopically, this is a high-grade carcinoma resembling ovarian HGSC. It shows a complex papillary pattern with fibrovascular cores and slit-like spaces or gaping glands. At high-power view, it has severe nuclear atypia, pleomorphism, hyperchromasia, and abundant mitoses (Fig. 17). The background endometrium on occasions shows the precursor lesion, known as serous endometrial intraepithelial carcinoma (Fig. 18A). This is characterized by high-grade atypical endometrial glands, frequently in the background of atrophy. Even in the absence of invasive carcinoma, the intraepithelial lesion can metastasize to extrauterine locations. Psammoma bodies, hob-nailing, and clear cells are frequently seen.^9^

**FIGURE 19:**
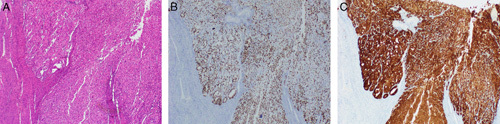
Uterine serous carcinoma. A, Tumor shows fused glands and sheets of cells with high-grade nuclear features. B, Immunohistochemical staining for p53 in uterine serous carcinoma showing positive nuclear staining in >90% of the tumor cells. C, Immunohistochemical staining for p16 in uterine serous carcinoma showing block positivity defined as strong and diffuse nuclear and cytoplasmic staining.

Nearly all USCs harbor *TP53* mutation and show p16 overexpression (Figs. 19A–C). The majority of serous carcinomas are negative for ER and PR. WT1 is usually focally positive or negative in USC, while it is diffusely positive in extrauterine serous carcinoma.^1^ One study has shown insulin-like growth factor II mRNA binding protein 3 positivity in 90% of USC compared with negative or weak staining in other uterine malignancies. Thus, insulin-like growth factor II mRNA binding protein 3 may be an additional USC marker.^52^

Endometrial carcinomas with serous and nonserous components are called mixed carcinomas if the proportion of the second type is 5% or more. The proportion of the serous component in these tumors should be specified as this may affect the prognosis. Many studies have shown common mutations in both components suggesting an origin from a single clone.^53^ The survival outcome of patients with mixed endometrial carcinoma may be less than that of endometrial carcinomas and similar to that of USC.^54^

The recent endometrial carcinoma classification, partly based on the molecular study, divides endometrial cancers into 4 subgroups. These are polymerase exonuclease (POLE) ultramutated, hypermutated microsatellite instability (mismatch repair deficient), copy-number low, and copy-number high (serous-like). This classification not only helps in cases with mixed histologic appearance but guides management and predicts prognosis. The TCGA study found that all USC are copy-number high. Other mutations identified in the serous-like subgroup were *CSMD3*, *TP53*, *PIK3CA*, *PTEN*, *PIK3R1*, *PPP2RIAm*, *FBXW7*, and *CHD4*.^55^

The PORTEC-3 trial showed a higher association of human epidermal growth factor receptor 2 (HER2) positivity with *TP53* mutation and serous histologic type. However, HER2 status did not have any independent prognostic value.^56^ Expression of HER2 was studied in USC, and it was found to vary from 14% to 80%. It was suggested that the wide range of HER2 scores reflect the lack of standardization in HER2 reporting. HER2-targeted treatment has shown to increase progression-free survival in HER2 amplified patients.^57^ Another clinically accessible target identified in USC is *PI3K3CA*. PI3K inhibitors have shown encouraging results in many solid tumors including endometrial carcinomas.^58^

USC may arise in a polyp in a background of an atrophic endometrium. The largest study of serous carcinoma arising in a polyp showed that although 60% of the cases had no residual tumor in the subsequent hysterectomy and ∼10% of the patients had a recurrence.^59^

Synchronous primary endometrial and ovarian cancers represent 10% of all endometrial and ovarian cancers and were previously considered independent entities. Though the majority of these are endometroid, around 10% of such cases were serous carcinomas. Clonality studies showed that most had a primary endometrial origin. Most of the USC and ovarian HGSC shared at least 1 somatic mutation and had unique mutations in addition to the shared mutations.^60^ Interestingly, while endometrial intraepithelial carcinoma (EIC) has been described as the precursor lesion of USC, serous tubal intraepithelial carcinoma (STIC) has been found in 6.5% of patients with USC compared with 0.1% in the general population.^61^

## PATHOGENESIS OF HIGH-GRADE SEROUS CARCINOMA

HGSCs of the female pelvis usually involve the ovary, fallopian tube, peritoneum, and endometrium. They constitute a group of highly complex and most perplexing diseases to understand, diagnose and treat. These tumors have challenged and defied researchers and medical professionals over the past 60 years. Patients with these neoplasms usually present with an advanced stage of the disease, so it is difficult to pinpoint the site of their origin. This is unlike cervical carcinoma and endometrial endometroid carcinoma, which are usually preceded by recognizable precursor lesions in the respective site. Most pelvic serous carcinomas have traditionally been assigned to an ovarian origin by default, primarily due to the location of the dominant bulk of the tumor rather than identifying a specific carcinogenic sequence or recognition of known precursor lesions. Furthermore, because most of these carcinomas involve the ovarian surface, an origin in the ovarian surface epithelium was presumed. Tumors without evidence of ovarian involvement were presumed to be primary in the peritoneal cavity.^62^,^63^

Epithelial ovarian cancers have traditionally been thought to arise from the ovarian surface epithelium, a modified mesothelium that lines the peritoneal cavity and covers most internal organs. A process of invagination and entrapment of the surface epithelium leads to the development of cortical inclusion cysts. Many of these cysts are lined by flattened epithelium resembling ovarian surface epithelium. The lining of these cysts is usually reactive for mesothelial IHC markers such as WT1 and calretinin. The second type of ovarian cortical cyst is lined by columnar cells with ciliated luminal borders resembling Müllerian duct epithelium.^62^,^63^ Developmentally, the Müllerian duct, is an anlage of female reproductive tract components, including fallopian tubes, uterus, the uterine cervix, and the superior portion of the vagina.^64^

**FIGURE 20:**
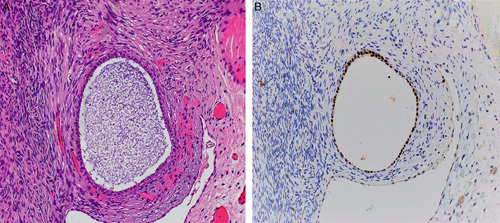
Benign cortical cyst in the ovary. A, Photomicrograph of a benign cortical cyst lined by secretory and ciliated cells. B, Immunohistochemistry staining for PAX8 in cortical cyst showing diffuse positivity in the lining epithelium.

The Müllerian-type ovarian cortical cysts may evolve from mesothelial cysts due to metaplasia triggered and supported by the surrounding ovarian stroma. Alternatively, these cysts may arise from an extraovarian source such as fallopian tube epithelium or Müllerian-type epithelial lining of para-ovarian and para-tubal cysts, endosalpingiosis, endometriosis, and endocervicosis (so-called “secondary Müllerian system”).^65^ These cysts are immunoreactive for PAX8 and usually negative for calretinin (Figs. 20A, B).

Ovarian cortical cysts with Müllerian epithelial lining seem to develop after puberty following rupture of the ovarian capsule during ovulation and with the entrapment of epithelium probably from the secondary Müllerian structures or fimbriated end of the fallopian tube. Furthermore, the relative proportion of Müllerian type of ovarian cortical cysts increases after menopause, suggesting that at least a subset of these cysts may indeed evolve because of Müllerian metaplasia in preexisting cortical cysts originally lined by ovarian surface type epithelium.^65^–^68^

The progression of Müllerian-lined cysts to serous neoplasms such as serous cystadenoma, adenofibroma, borderline serous tumors as well as LGSC is well documented and accepted. However, examples of the transition from these benign cystic structures to HGSC are rare.^69^ These observations are arguments against the concept that HGSCs originate from ovarian cortical cysts.

A revolutionary change in our understanding of the pathogenesis of HGSC has occurred over the last 2 decades.^70^ It all began with the discovery of *BRCA1* and *BRCA2* tumor suppressor genes. Approximately 5% to 10% of ovarian cancers are due to inherited germline mutations of susceptible genes, and about 90% of such cases involve mutations of *BRCA1* or *BRCA2* genes. Mutation carriers have an increased risk of ovarian cancer (40% to 60%) at the age of 70 years, compared with the lifetime risk of the general population (1.3%). Women with hereditary ovarian cancer syndrome undergo risk-reducing bilateral salpingo-oophorectomy. At the beginning of 2000, there were several reports of histologic findings in these specimens. Surprisingly, epithelial abnormalities suggestive of precursor lesions of HGSC were found predominantly in the fallopian tubes rather than the ovaries.^70^–^74^ In 2005, Brigham and Women’s Hospital group introduced the Sectioning and Extensively Examining the Fimbriated End of the Fallopian Tube (SEE-FIM) protocol for the routine analysis of fallopian tubes from women with *BRCA* mutations.^75^,^76^ This protocol triggered increased reports of early serous carcinoma and other related lesions in ∼2% of the cases, mainly in the fimbriae of the fallopian tubes. This discovery led to the hypothesis that serous carcinoma in the ovary or other pelvic sites originates from the fallopian tubes. Furthermore, with the use of SEE-FIM protocol, ∼50% of patients with HGSC were found to have these lesions. The frequency of these lesions in subsequent studies varied from 20% to 60%.

**FIGURE 21:**
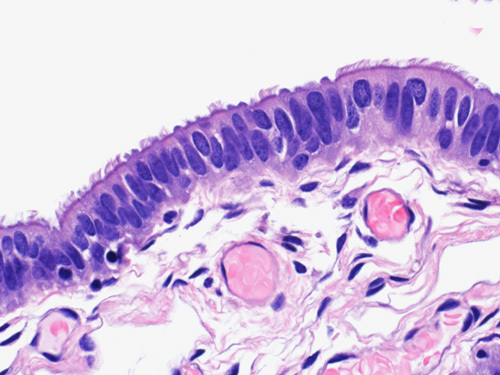
Histologic image of a normal fallopian tube featuring pseudostratified lining composed of ciliated and secretory cells.

The normal epithelial lining of the fallopian tubes is composed of a variable mixture of ciliated and secretory cells (Fig. 21). This epithelium is exposed to various inflammatory stimuli from fluid released from ruptured ovarian follicles and due to retrograde menstruation. The cells exposed to these harmful stimuli, especially at the fimbriated part of the fallopian tubes, may sustain DNA damage, setting in motion a series of changes resulting in cancer precursor lesions and ultimately leading to cancer.

**FIGURE 22:**
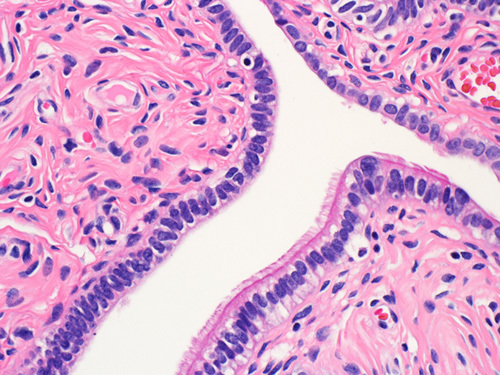
Secretory cell outgrowth in a fallopian tube characterized by secretory cell predominance in the lining epithelium. Notice the normal ciliated epithelium in the lower right corner.

The development of morphologic abnormalities starts with patches of epithelium lined predominantly by secretory cells instead of the more differentiated ciliated cells. These changes may be seen in any part of the fallopian tube and are called secretory cell outgrowth (Fig. 22).^77^,^78^

**FIGURE 23:**
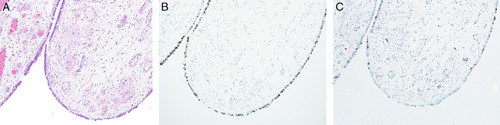
p53 signature in a fallopian tube. A, Fallopian tube epithelium showing increased cellularity and dysplasia in a case of p53 signature. B, Immunohistochemical stain for p53 showing positivity in most of the cells indicating p53 signature. C, Ki-67 immunohistochemistry in a case of p53 signature showing positivity in a few cells only.

Prolonged and repeated exposure of the fallopian tube secretory cells to inflammatory cytokines may cause intracellular inflammation and genetic stress, leading to DNA damage and *TP53*-modulated apoptosis. Over time, some of these cells may acquire *TP53* mutation but without manifesting excessive cellular proliferation. These cells are strongly immunoreactive for p53, and the resulting lesion is designated as p53 signature, defined as 12 or more consecutive p53-positive normal-appearing secretory tubal epithelial cells (Figs. 23A–C). This lesion is predominantly located at the distal part of the fallopian tube and is a nonobligatory precursor of carcinoma. p53 signature may be present in multiple foci in both healthy and *BRCA*-mutated fallopian tubes.^79^–^82^

**FIGURE 24:**
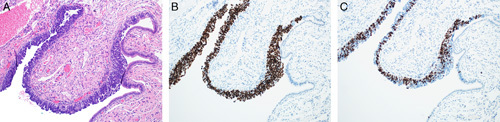
Serous tubal intraepithelial carcinoma. A, Serous tubal intraepithelial carcinoma showing cellular proliferation, nuclear hyperchromasia, pleomorphism and loss of polarity. The adjacent normal epithelium can be appreciated with a single cell lining of ciliated epithelium without atypia. B, Immunohistochemistry for p53 in a serous tubular in situ carcinoma lesion showing strong diffuse nuclear staining. The adjacent normal epithelium has a wild-type staining (focal weak positivity). C, Ki-67 immunohistochemistry with >60% of cells showing positive staining in contrast to the adjacent normal epithelium with only 2% proliferative index.

Progression of p53 signature to a neoplastic process involves acquiring additional genetic alterations, such as mutations or hypermethylation in *BRCA1/BRCA2*, elevated stathmin, shortened telomeres, and cyclin E amplification. The resulting lesion is an intraepithelial neoplasm termed STIC which manifests increased cell proliferation, nuclear hyperchromasia, pleomorphism, loss of polarity, and increased mitotic activity (Fig. 22). In addition to being positive for p53, these lesions also reveal a high proliferation index demonstrated by Ki-67 staining (Figs. 24B, C). In some cases, the p53 signature may harbor a nonsense mutation of p53. These lesions stain negative for p53 but may be recognized by IHC staining for alpha HIA, a marker for DNA damage.^79^–^82^

**FIGURE 25:**
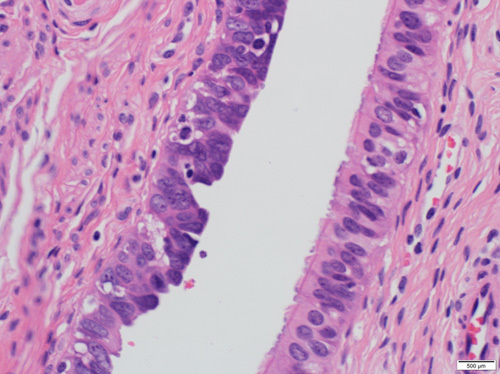
Normal fallopian tube lining epithelium (right) adjacent to serous tubular in situ carcinoma with hypercellularity, hyperchromasia and nuclear atypia.

**FIGURE 26:**
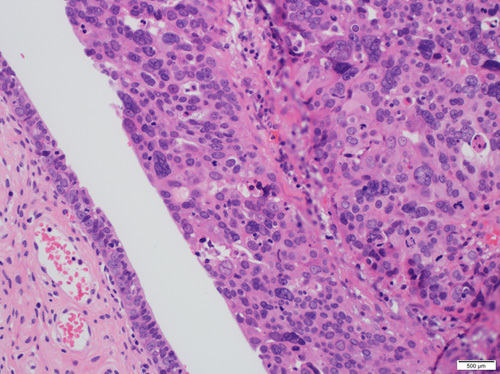
Serous tubal intraepithelial carcinoma (left) adjacent to a high-grade serous carcinoma in a fallopian tube. Similar histology can be appreciated in both lesions with the carcinoma showing a higher degree of atypia and bizarre nuclei.

**FIGURE 27:**
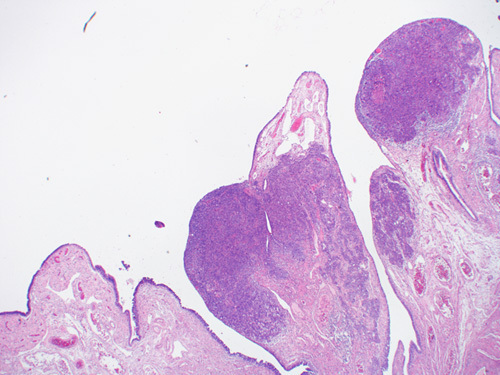
Histologic image of high-grade serous carcinoma arising from serous tubular in situ carcinoma in the fimbriated end of a fallopian tube.

**FIGURE 28:**
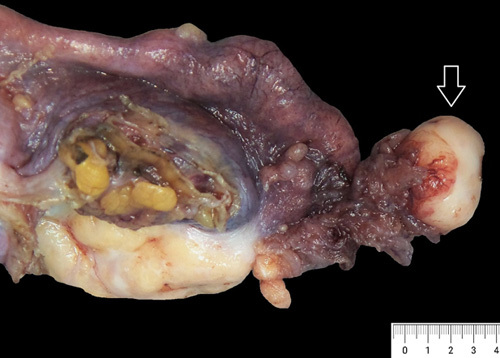
Gross photograph of tubal serous carcinoma (arrow). The fimbriated end can be seen adherent to the tumor.

Malignant cells from STIC lesions may invade the fallopian tube wall and progress to invasive carcinoma of the fallopian tube (Figs. 25–28). Alternatively, the malignant cells from STIC can implant onto the ovarian surface and progress to an ovarian HGSC. Cancer cells from STIC may also exfoliate into the peritoneal cavity and later give rise to peritoneal/omental HGSC. Thus, according to the current thinking, most pelvic HGSCs have an origin in the epithelium of the fallopian tube. The crucial role of STICs in the genesis of pelvic HGSC is supported by targeted sequencing studies that have revealed identical *TP53* mutation in the premalignant tubal lesions as in the HGSC in various locations in the pelvis.^83^

Cases of HGSC arising in the absence of STIC have also been reported suggesting that other, yet unidentified, precursor lesions might exist. Based on multiple studies, the frequency of an intramucosal carcinoma in the fallopian tube in patients with symptomatic or advanced HGSC varies from 10% to 60%, leaving a large percentage of HGSC in which an early malignancy in the fallopian tube was not identified. The WHO’s recent publication has proposed criteria to assign the primary site of origin when a detailed workup fails to identify definite precursor lesions. According to this scheme, a tumor in the ovary may be considered primary ovarian if no definite STIC or intramucosal carcinoma can be shown in the fallopian tube, and uterine HGSC has been excluded. Similarly, a peritoneal carcinoma without an ovarian or fallopian tube or uterine primary may be presumed to arise in the peritoneum.^1^ Clear evidence to support this concept, however, is lacking in the published literature.

An interesting study by Soong and colleagues may shed more light on this topic. During an exhaustive analysis of seemingly “normal” fallopian tubes of 32 women with HGSC, very early signs of precancerous cells (which the investigators termed “early serous proliferations,” or ESPs) were found in 13 women. Suspecting that some ESPs might fuel the development of HGSC, the investigators sequenced *TP53* in the fallopian tubes of the 13 women with HGSC but without evidence of STIC. In 12 of the 13 women studied, the team detected a mutation in the ESPs that matched the mutation in cancer found in the pelvis. Based on these findings, the investigators concluded that some ESPs exit the fallopian tubes, survive at another location, and eventually evolve into HGSC. They coined a term for this phenomenon: precursor escape. According to this concept, cells with mutations in *TP53* and other genes may not appear malignant in a morphologic sense. Still, they may have the capacity to survive and proliferate in another location, such as the peritoneum or ovary, and after a variable interval, give rise to HGSC. However, in the fallopian tube, these cells may not have progressed to STIC. This may explain the development of advanced carcinoma in the ovary and peritoneum without evidence of STIC in the fallopian tube.^84^

The concept of “precursor escape” as one of the mechanisms of development of HGSC, although attractive, requires validation by additional studies. Furthermore, a precise characterization of ESPs regarding morphologic features, IHC staining pattern, and genetic profile is yet to be established.

### Pathogenesis of Serous Carcinoma of the Endometrium

Pathogenesis of serous carcinoma of the endometrium is closely intertwined with that of HGSC in general. Several precursor lesions manifesting different stages of cancer evolution are recognized. These include p53 signature, endometrial glandular dysplasia, and EIC. *TP53* mutation is an initial but essential molecular abnormality involved in the development and progression of this tumor, and tumor cells usually are strongly positive for p53 immunostaining. The lesions of the p53 signature appear similar to those seen in the fallopian tube and consist of clusters of normal-appearing cells with strong immunostaining for p53.^83^,^85^

Endometrial intraepithelial carcinoma may be present in isolation or, more commonly, adjacent to a focus of invasive carcinoma. It is usually present near the surface of the endometrium, replacing the surface endometrial epithelium with variable extension into underlying glands. The lesion consists of highly atypical cells cytologically identical to those comprising the invasive serous carcinoma. Endometrial intraepithelial carcinoma may be associated with the high-stage disease with involvement of the ovaries and peritoneum and a fatal outcome. The widespread tumor dissemination is probably due to the malignant cells from the EIC traveling to the peritoneal cavity and ovary through the fallopian tube and giving rise to metastatic disease in these locations. This peritoneal and ovarian spread may be seen even without myometrial invasion.^34^,^86^

A small subgroup of patients with USC may also have STIC lesions, suggesting that STIC may on occasion act as the precursor for USC and that malignant cells from STIC may travel from the fallopian to the uterine cavity and give rise to USC. However, some authors have suggested that these STICS are metastatic implants from USC rather than precursor lesions for uterine cancer.^87^
